# Prediction of regulatory targets of alternative isoforms of the epidermal growth factor receptor in a glioblastoma cell line

**DOI:** 10.1186/s12859-019-2944-9

**Published:** 2019-08-22

**Authors:** Claus Weinholdt, Henri Wichmann, Johanna Kotrba, David H. Ardell, Matthias Kappler, Alexander W. Eckert, Dirk Vordermark, Ivo Grosse

**Affiliations:** 1 Institute of Computer Science, Martin Luther University Halle–Wittenberg, Halle, Germany; 2 Department of Oral and Maxillofacial Plastic Surgery, Martin Luther University Halle–Wittenberg, Halle, Germany; 3 Institute for Molecular and Clinical Immunology, Otto-von-Guericke-University, Magdeburg, Germany; 4 Molecular Cell Biology, School of Natural Sciences, University of California, Merced, USA; 5 Department of Radiotherapy, Martin Luther University Halle–Wittenberg, Halle, Germany; 6 German Center of Integrative Biodiversity Research (iDiv) Halle-Jena-Leipzig, Leipzig, Germany

**Keywords:** EGFR, Splice variants, RNAi, Bayesian Information Criterion, Bayesian Gene Selection Criterion

## Abstract

**Background:**

The epidermal growth factor receptor (EGFR) is a major regulator of proliferation in tumor cells. Elevated expression levels of EGFR are associated with prognosis and clinical outcomes of patients in a variety of tumor types. There are at least four splice variants of the mRNA encoding four protein isoforms of EGFR in humans, named I through IV. EGFR isoform I is the full-length protein, whereas isoforms II-IV are shorter protein isoforms. Nevertheless, all EGFR isoforms bind the epidermal growth factor (EGF). Although EGFR is an essential target of long-established and successful tumor therapeutics, the exact function and biomarker potential of alternative EGFR isoforms II-IV are unclear, motivating more in-depth analyses. Hence, we analyzed transcriptome data from glioblastoma cell line SF767 to predict target genes regulated by EGFR isoforms II-IV, but not by EGFR isoform I nor other receptors such as HER2, HER3, or HER4.

**Results:**

We analyzed the differential expression of potential target genes in a glioblastoma cell line in two nested RNAi experimental conditions and one negative control, contrasting expression with EGF stimulation against expression without EGF stimulation. In one RNAi experiment, we selectively knocked down *EGFR* splice variant I, while in the other we knocked down all four *EGFR* splice variants, so the associated effects of *EGFR* II-IV knock-down can only be inferred indirectly. For this type of nested experimental design, we developed a two-step bioinformatics approach based on the Bayesian Information Criterion for predicting putative target genes of EGFR isoforms II-IV. Finally, we experimentally validated a set of six putative target genes, and we found that qPCR validations confirmed the predictions in all cases.

**Conclusions:**

By performing RNAi experiments for three poorly investigated EGFR isoforms, we were able to successfully predict 1140 putative target genes specifically regulated by EGFR isoforms II-IV using the developed Bayesian Gene Selection Criterion (BGSC) approach. This approach is easily utilizable for the analysis of data of other nested experimental designs, and we provide an implementation in R that is easily adaptable to similar data or experimental designs together with all raw datasets used in this study in the BGSC repository, https://github.com/GrosseLab/BGSC.

**Electronic supplementary material:**

The online version of this article (10.1186/s12859-019-2944-9) contains supplementary material, which is available to authorized users.

## Background

Glioblastoma is the most malignant and most frequent primary cerebral tumor in adults and is responsible for 65% of all brain tumors [1]. One potential molecular target amplified in 36% of glioblastoma patients is the epidermal growth factor receptor (EGFR), and the expression of EGFR is associated with prognosis in cancer [2]. EGFR is known to affect growth and survival signals and to play a crucial role in the regulation of cell proliferation, differentiation, and migration of various tumor entities [3]. Hence, EGFR is well known as a prognostic tumor marker and therapeutic target in different tumor entities.

The full-length transmembrane glycoprotein isoform of EGFR consists of three functional domains of which the extracellular domain is capable of binding at least seven different ligands such as EGF, AREG, or TGF- *α* [4]. However, there are at least three different truncated *EGFR* splice variants (II, III, and IV). Up to now, only the full-length EGFR isoform I translated from *EGFR* splice variant I is well investigated, but comparatively little is known about the biological significance of the truncated EGFR isoforms II-IV translated from *EGFR* splice variants II-IV.

EGFR isoforms II-IV lack the intra-cellular tyrosine-kinase domain [5], and Maramotti et al. [6] describes that EGFR isoforms II-IV can potentially function as natural inhibitors of EGFR isoform I. EGFR isoforms II-IV bind EGF with similar binding kinetics but lower binding affinity than EGFR isoform I [7], which binds EGF with a dissociation constant of 1.77×10^−7^*M* [8].

Different tumor therapies targeting EGFR via antibodies or small molecules often do not have response rates as successful as expected. EGFR isoforms II-IV may be responsible for therapeutic failures because they do not contain the tyrosine-kinase domain targeted by small molecules. However, they do contain the extracellular N-terminus of EGFR, which is bound by therapeutic antibodies. Nevertheless, EGFR-specific antibody therapy requires the interaction of EGFR-bound therapeutic antibodies with presenting cells. EGFR isoforms II-IV are soluble proteins that do not mark the expressing cell itself, but rather diffuse in the extracellular space, probably bind to surrounding non-tumor cells, and possibly mislead the immune system.

This problem motivated the present work of perturbing the profile of the four *EGFR* splice variants using small interfering RNAs (siRNAs) that differentially target these splice variants and of measuring the resulting expression responses using traditional microarrays. It is impossible to knock-down only *EGFR* splice variants II-IV and not *EGFR* splice variant I by RNAi because there is no region specific to only *EGFR* splice variants II-IV. Hence, we performed the RNAi experiments according to the nested experimental design as shown in Table 1. Based on this design, the associated effects of a knock-down of *EGFR* splice variants II-IV can only be inferred indirectly by subtracting the effects found by knocking down only *EGFR* splice variant I from the effects found by knocking down all *EGFR* splice variants I-IV. The problem of only indirectly measurable gene regulation or receptor effects of nested splice variants is widespread in many regulatory pathways and many species, so we developed a two-step bioinformatics approach for the prediction of putative target genes called Bayesian Gene Selection Criterion (BGSC) approach, which we tested by quantitative real-time polymerase chain reaction (qPCR) experiments.

**Table 1:** Experimental design where the rows present the RNAi treatment – without RNAi, RNAi against EGFR splice variant I (siRNA_*I*_), and RNAi against all EGFR splice variants (siRNA_*ALL*_) – and the columns present the EGF treatment

	no EGF	EGF
no RNAi	*x*_1_	*x*_2_
RNAi by siRNA_*I*_	*x*_3_	*x*_4_
RNAi by siRNA_*ALL*_	*x*_5_	*x*_6_

The six corresponding logarithmic expression values per gene are denoted by *x*_1_,…,*x*_6_

The rest of this paper is structured as follows: In Results, we describe the identification of a cell line with an inducible EGFR-signaling pathway, investigate the specificity of siRNAs, introduce the two-step BGSC approach for predicting putative target genes regulated by EGF via EGFR isoforms II-IV and not by the full-length EGFR isoform I or other receptors, and describe the qPCR validation experiments. In Discussion, we discuss the adjustability of the EGFR-signaling pathway in cell line SF767 and the biological relevance of the validated genes.

## Results

### Identification of a cell line with an inducible EGFR-signaling pathway

**Fig. 1:**
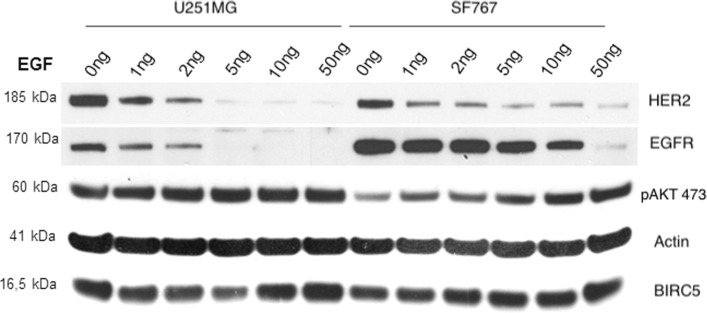
Western blot analysis of the two glioblastoma cell lines U251MG and SF767. U251MG is a PTEN mutant and PIK3CA wild-type cell line and SF767 is a PTEN wild-type and PIK3CA (E545K) mutant cell line. Cells were treated for 24 hours with different levels of the EGFR-ligand EGF (0-50 ng/ml). The levels of HER2 and EGFR are reduced by EGF-dependent degradation of the formed and internalized EGF-HER2/EGFR complexes. The activation of AKT-protein (phosphorylation of the Ser473) is detectable in an EGF-dependent manner in cell line SF767, whereas the pAKT level is constantly high in cell line U251MG. These observations indicate that the EGFR-signaling pathway is inducible in cell line SF767, but not in cell line U251MG. Anti- *β*-actin staining was done as a loading control, and BIRC5 (survivin) was used as an indicator for proliferation activity

A meaningful analysis of the EGFR-signaling pathway is possible only in a cell line with an adjustable pathway, e.g., by a response to ligand stimulation or treatment by a tyrosine kinase inhibitor (TKI) [9]. Hence, we investigated four glioblastoma cell lines in a pilot study to identify a cell line with an adjustable EGFR-signaling pathway. Figure 1 shows the measured protein levels of phosphorylated AKT (pAKT) resulting from the treatment of two of these cell lines U251MG and SF767 with increasing levels of recombinant ligand EGF. We found that the pAKT (Ser473) level in cell line U251MG is constantly high, possibly resulting from the mutated *PTEN* gene [10]. In the *PTEN* wild-type cell line SF767 [11], pAKT showed a level of activity even without adding recombinant EGF due to the E545K-mutation of gene *PIK3CA* present in this cell line [12]. However, the activity of pAKT could be increased three-fold by adding recombinant EGF as a ligand, indicating that the EGFR-AKT signaling pathway was inducible in an EGF-dependent manner (Fig. 1). Figure 1 also shows that the full-length EGFR protein disappeared by applying a high concentration of EGF of 50 ng/ml to cell line SF767. This high concentration of EGF leads to the saturation of the full-length EGFR protein with the ligand EGF, to the subsequent internalization and degradation of the formed EGF-EGFR complex, and thus to the observed disappearance of the full-length EGFR protein.

### Specificity of siRNAs

**Fig. 2:**
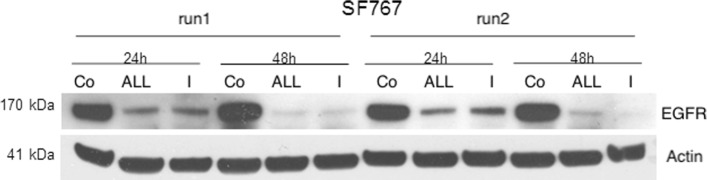
Western blot analysis of the effect of the two different siRNAs. Knock-down of the EGFR full-length protein level using two different siRNA constructs (*siRNA*_*ALL*_ and *siRNA*_*I*_). Both siRNA constructs reduce the full-length EGFR protein level at 24 hours and 48 hours after the start of the experiment, while the Actin level is not affected

We performed RNAi experiments with a siRNA against *EGFR* splice variant I, henceforth called siRNA_*I*_ and with a siRNA against all *EGFR* splice variants, henceforth called siRNA_*ALL*_ (Table 2). To investigate the specificity of the two siRNA constructs siRNA_*ALL*_ and siRNA_*I*_, we analyzed mRNA levels and protein levels of EGFR. Figure 2 shows that the treatment of SF767 cells with the two siRNAs reduced the level of full-length EGFR protein 24 hours and 48 hours after the start of the experiment. We then analyzed the siRNA-specificity by qPCR experiments for (a) all *EGFR* splice variants together, (b) *EGFR* splice variant I (full-length), (c) *EGFR* splice variant IV, and (d) the two genes *MMP2* and *GAPDH* as a control. Additional file 1: Figure S.1 shows that the application of siRNA_*ALL*_ and siRNA_*I*_ reduced the levels of all *EGFR* splice variants by 70.9*%* on average and the levels of the full-length *EGFR* splice variant I by 78.1*%* on average. Additional file 1: Figure S.1 also shows that the application of siRNA_*ALL*_ reduced the levels of *EGFR* splice variant IV by 69.9*%* on average, that the application of siRNA_*I*_ did not reduce the levels of *EGFR* splice variant IV, and that the application of siRNA_*ALL*_ and siRNA_*I*_ did not reduce the levels of the two control genes.

**Table 2:** Design of siRNA_*ALL*_, siRNA_*I*_, and nonsense siRNA

siRNA	sequence 5^′^→3^′^	target mRNA	localization	corresponding mRNA
I	AACGCAUCCAGCAAGAAUA	EGFR I	4098-4116	NM_005228.3
ALL	CGGAAUAGGUAUUGGUGAA	EGFR I	1260-1278	NM_005228.3
		EGFR II		NM_201282.1
		EGFR III		NM_201283.1
		EGFR IV		NM_201284.1
nonsense	CGTACGCGGAATACTTCGA			

### First step of the BGSC approach - grouping of genes

**Fig. 3:**
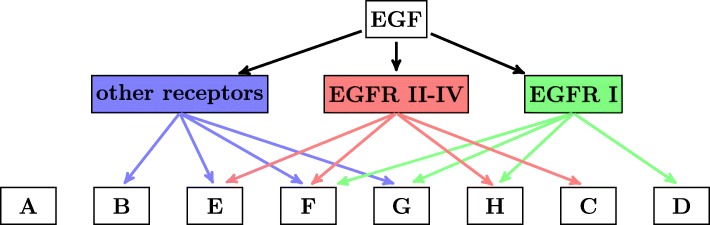
Graphical representation of the eight gene groups. Each gene can be transcriptionally regulated by some combination of *EGFR* splice variant I (green arrows), *EGFR* splice variants II-IV (red arrows), and other EGF receptors (blue arrows), resulting in eight gene groups A - H

The binding affinities of the three EGFR isoforms II-IV to EGF are lower than that of the full-length EGFR isoform I [7] and probably different from each other, but yet very high [7], so we assume that the high concentration of EGF of 50 ng/ml leads to the saturation of all EGFR isoforms irrespective of their different binding affinities to EGF. Hence, we make the simplifying assumption here and in the following that the concentration of the ligand is sufficiently high for neglecting the binding affinities of the four EGFR isoforms I-IV to EGF. Under this simplifying assumption, we define groups with distinct expression patterns considering all eight possible modes of EGF-triggered transcriptional gene regulation via EGFR isoform I, via EGFR isoforms II-IV, or via other non-EGF receptors, and we observe that each gene can be grouped into exactly one of the following eight gene groups A - H, which are graphically represented by Fig. 3:

- Group A contains genes not regulated by EGF.
- Group B contains genes regulated by EGF not via EGFR isoforms I-IV, but via other receptors.
- Group C contains genes regulated by EGF via EGFR isoforms II-IV and not via EGFR isoform I and not via other receptors.
- Group D contains genes regulated by EGF via EGFR isoform I and not via EGFR isoforms II-IV and not via other receptors.
- Group E contains genes regulated by EGF via EGFR isoforms II-IV and via other receptors and not via EGFR isoform I.
- Group F contains genes regulated by EGF via EGFR isoform I and via EGFR isoforms II-IV and via other receptors.
- Group G contains genes regulated by EGF via EGFR isoform I and via other receptors and not via EGFR isoforms II-IV.
- Group H contains genes regulated by EGF via EGFR isoform I and via EGFR isoforms II-IV and not via other receptors.

**Fig. 4:**
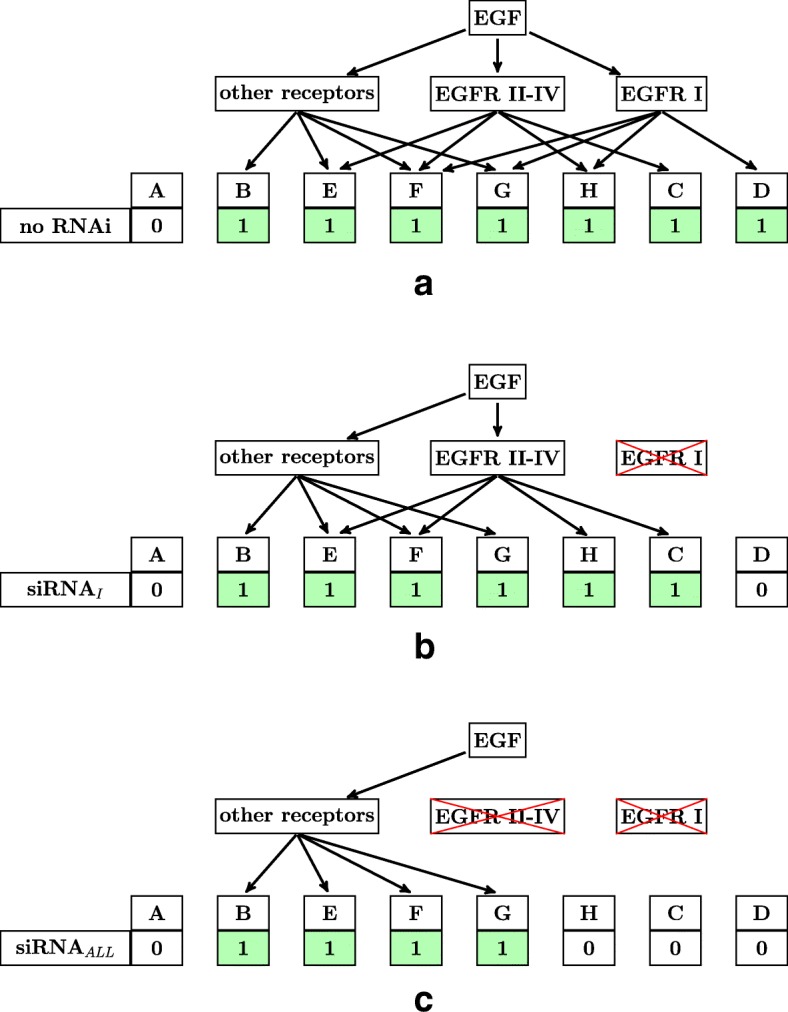
Graphical representation of EGF regulation by RNAi treatment. Each differentially expressed gene can be grouped into exactly one of the following eight gene groups A - H. These eight gene groups (A - H) contain all possible theoretical models of regulation of a gene, after EGF addition in combination with the three RNAi treatments. Subfigure (**a**) corresponds to the control experiment without RNAi treatment, subfigure (**b**) corresponds to RNAi treatment with siRNA_*I*_, and subfigure (**c**) corresponds to RNAi treatment with siRNA_*ALL*_. Red crosses indicate the down-regulation of EGFR by RNAi treatment with siRNA_*I*_ (**b**) or siRNA_*ALL*_ (**c**). The change of gene expression (up or down) by EGF treatment is indicated by 1 and no change by 0, i.e., all genes except those of gene group A should be differentially expressed in the control experiment (**a**), all genes except those of gene groups A and D should be differentially expressed in experiment (**b**), and all genes except those of gene groups A, C, D, and H should be differentially expressed in experiment (**c**)

Next, we consider for each RNAi treatment if the genes of each group would be differentially regulated after EGF-stimulation. To conceptually analyze the gene expression of each group we denote by ~1~ a theoretical regulation (up or down) of the group after addition of EGF and denote by ~0~ no regulation. Further, we define groups as regulated after EGF-stimulation if there is at least one incoming edge to the group in the graphical representation (Fig. 4), and we define groups with no incoming edge as unregulated. We consider three experimental manipulations with RNAi: negative control without RNA interference, RNAi with siRNA against *EGFR* splice variant I, henceforth called siRNA_*I*_, and RNAi with siRNA against all *EGFR* splice variants, henceforth called siRNA_*ALL*_ (Fig. 4).

First, we consider the negative control without RNA interference (Fig. 4a). Here, none of the *EGFR* splice variants are down-regulated by a siRNA, so all target genes of EGFR isoforms and target genes of other EGF receptors can be induced by EGF. Hence, we expect differential expression under EGF stimulation of genes belonging to groups B - H on the one hand and no differential expression of genes belonging to group A on the other hand.

Second, we consider RNAi treatment with siRNA_*I*_ (Fig. 4b). Here, only *EGFR* splice variant I is down-regulated by siRNA_*I*_, so only target genes of EGFR isoforms II-IV and target genes of other EGF receptors can be induced by EGF. Hence, we expect differential expression by EGF treatment of genes belonging to groups B, C, and E - H on the one hand and no differential expression of genes belonging to groups A and D on the other hand.

Third, we consider RNAi treatment with siRNA_*ALL*_ (Fig. 4c). Here, all four *EGFR* splice variants are down-regulated by siRNA_*ALL*_, so only target genes of other EGF receptors can be induced by EGF. Hence, we expect differential expression by EGF treatment of genes belonging to groups B and E - G on the one hand and no differential expression of genes belonging to groups A, C, D, and H on the other hand.

**Fig. 5:**
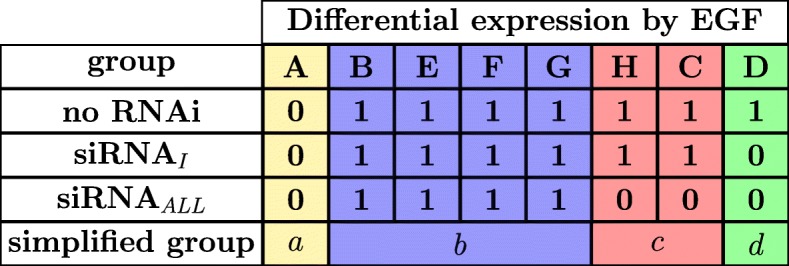
Reduction of the conceptual gene groups. Genes of group A are never differentially expressed by EGF treatment. Genes of group B and E - G are always differentially expressed by EGF treatment. Genes of group C and H are differentially expressed by EGF treatment in case of control treatment (no RNAi) or simultaneous treatment with siRNA_*I*_, whereas not differentially expressed by EGF treatment in case of simultaneous treatment with siRNA_*ALL*_. Genes of group D are differentially expressed by EGF treatment in case of control treatment (no RNAi), whereas not differentially expressed by EGF treatment in case of simultaneous treatment with siRNA_*I*_ or siRNA_*ALL*_. We find that the eight gene groups show only four different expression patterns, so we reduce the eight gene groups A - H to the four simplified gene groups *a* - *d*, where group A becomes group *a*, the union of the groups B and E - G becomes group *b*, the union of the groups C and H becomes group *c*, and group D becomes group *d*

Figure 5 summarizes the different expression patterns of Fig. 4. We find that the eight gene groups show only four different expression patterns, so we reduce the eight gene groups A - H to the four simplified gene groups *a* - *d*, where group A becomes group *a*, the union of the groups B and E - G becomes group *b*, the union of the groups C and H becomes group *c*, and group D becomes group *d*.

These simplified gene groups can be easily interpreted as follows: Genes of group *a* are not regulated by EGF, whereas genes of groups *b*−*d* are regulated by EGF. Genes of group *b* are regulated by EGF only through other receptors besides EGFR isoforms. Genes of group *c* are regulated by EGFR isoforms II-IV and not by other receptors. And genes of group *d* are regulated by EGFR isoform I and not by EGFR isoforms II-IV or other receptors. Based on this reduction, we can now formulate the goal of this work as the prediction of putative target genes regulated by EGFR isoforms II-IV and not by other receptors or, more crisply, as the goal of predicting genes of group *c*.

### Second step of the BGSC approach - classification of genes

In the second step, we classify each potential target gene into one of the four simplified gene groups *z* ∈ {*a, b*,*c, d*} based on the Bayesian Information Criterion, and thereby predict target genes regulated by EGF via EGFR isoforms II-IV as those classified into group *c*.

In this step, we apply the oversimplified, but commonly accepted, assumption that the log-transformed expression of each gene is normally distributed [13] with a gene-specific and treatment-specific mean and variance.

**Fig. 6:**
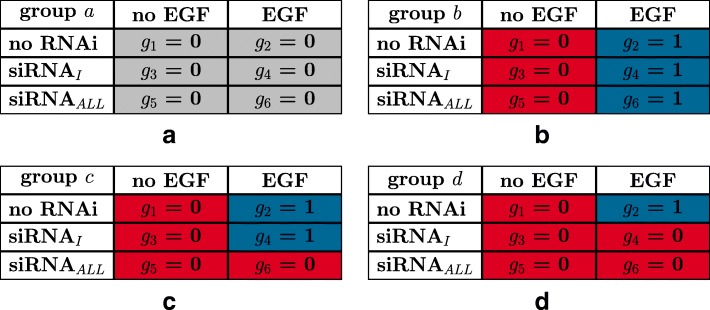
Schematic expression patterns. For gene groups **b** – **d** (Subfigures **b** – **d**) the indicator variables *g*_*n*_ are equal to 0 if the logarithmic expression levels *x*_*n*_ are expected to be similar to *x*_1_ and 1 otherwise (Table 1). The four no-EGF columns are equal to 0 by model assumption 1, and the four EGF columns are equal to the corresponding columns of Fig. 5 by model assumption 2. For gene group a (Subfigure **a**) the indicator variables *g*_*n*_ are equal to 0 by definition

For each gene, we additionally assume heteroscedasticity, i.e., equality of the six variances, of the six normally distributed logarithmic expression values under each of the six experimental conditions, an assumption commonly made in the *t*-test, the analysis of variance, or other statistical tests. We further assume that the six means of these six normal distributions are group specific as shown in Fig. 6.

First, we assume genes of group *a* (not regulated by EGF) to show no differential expression under each of the six experimental treatments (Table 1), as manifested by equality of the six means of the six normal distributions (Fig. 5, yellow column).

Second, we assume genes of group *b* (regulated by EGF through other receptors besides any EGFR isoform) to show differential expression under EGF-stimulation, irrespective of RNAi treatment targeting any EGFR isoform (Fig. 5, blue column). Hence, we assume genes of group *b* to have two different mean logarithmic expression levels, one in samples 1, 3, and 5, and another potentially different one in samples 2, 4, and 6 (Table 1). We denote these two mean logarithmic expression levels by *μ*_*b*0_ (Fig. 6b red) and *μ*_*b*1_ (Fig. 6b blue) respectively.

Third, we assume genes of group *c* (regulated by EGFR isoform II-IV and not by other receptors) to show differential expression between the negative control and siRNA_*ALL*_ treatments (Fig. 5, red column) under EGF-stimulation. Hence, we assume genes of group *c* to have two different mean logarithmic expression levels, one in samples 1, 3, 5, and 6, and another potentially different one in samples 2 and 4 (Table 1). We denote these two mean logarithmic expression levels by *μ*_*c*0_ (Fig. 6c red) and *μ*_*c*1_ (Fig. 6c blue) respectively.

Fourth, we assume genes of group *d* (regulated by EGFR isoform I only) to show differential expression between the negative control and siRNA_*I*_ treatment (Fig. 5, green column) under EGF-stimulation. Hence, we assume genes of group *d* to have two different mean logarithmic expression levels, one in samples 1, 3, 4, 5, and 6, and another potentially different one in sample 2 (Table 1). We denote these two mean logarithmic expression levels by *μ*_*d*0_ (Fig. 6d red) and *μ*_*d*1_ (Fig. 6d blue) respectively.

For genes of group *a* we denote the two model parameters *μ*_*a*_ and *σ*_*a*_ of the six normal distributions by *θ*_*a*_=(*μ*_*a*_,*σ*_*a*_), and for each of the three groups $\tilde z \in \{b,c,d\}$ we denote the three model parameters $\mu _{\tilde z0}$, $\mu _{\tilde z1}$, and $\sigma _{\tilde z}$ of the six normal distributions by $\theta _{\tilde z} = (\mu _{\tilde z0}, \mu _{\tilde z1}, \sigma _{\tilde z})$.

Assuming conditional independence of the six logarithmic expression levels given group *z* and model parameters *θ*_*z*_, we can write the likelihood *p*(*x*|*z*,*θ*_*z*_) of data *x* given group *z* and model parameters *θ*_*z*_ as a product of six univariate normal distributions with the corresponding mean *μ*_*a*_, or means $\mu _{\tilde z0}$ and $\mu _{\tilde z1}$, and the corresponding variance $\sigma ^{2}_{z}$ (Eqs. 1 and 2). Using the maximum likelihood principle, we obtain the estimates of model parameters *θ*_*a*_ by Eqs. 8*a* and 8*b* and of model parameters $\theta _{\tilde z}$ for $\tilde z \in \{b,c,d\}$ by Eqs. 8*c*, 8*d* and 8*e*.

**Fig. 7:**
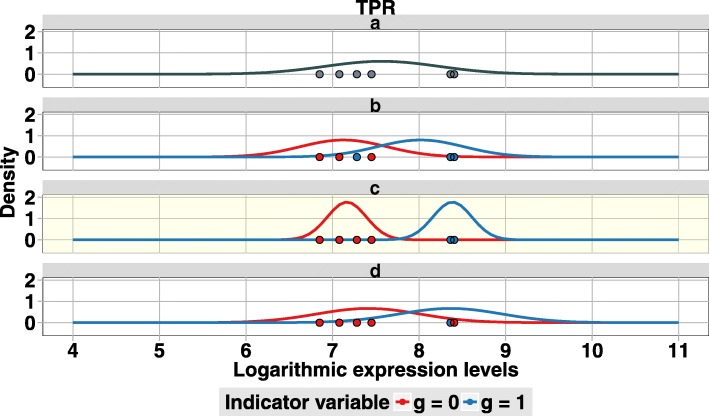
Probability density plot of the normal distributions of TRP. For group *a* we mark the logarithmic expression values *x*_1_,…,*x*_6_ of *TPR* with black points, which are colored according to Fig. 6a, and assume that all six logarithmic expression levels stem from the same normal distribution. In black, we plot the probability density of this normal distribution with mean and standard deviation equal to *μ* and *σ* of the six logarithmic expression levels. For groups *b* - *d* we assume that all six logarithmic expression levels stem from a mixture of two normal distributions with independent means *μ*_0_ and *μ*_1_ and one pooled standard deviation *σ*. We mark the logarithmic expression values *x*_1_,…,*x*_6_ of *TPR* with points which are colored according to indicator variables from Fig. 6 *g*=0 in red and *g*=1 in blue and we plot the probability densities of the two normal distributions in red and blue, respectively. For group *b* we assume that the logarithmic expression levels *x*_1_, *x*_3_, and *x*_5_ stem from the normal distribution with mean *μ*_0_ (red) and *x*_2_, *x*_4_, and *x*_6_ from the normal distribution with mean *μ*_1_ (blue). For gene group c we assume that the logarithmic expression levels *x*_1_, *x*_3_, *x*_5_, and *x*_6_ stem from the normal distribution with mean *μ*_0_ (red) and *x*_2_ and *x*_4_ from the normal distribution with mean *μ*_1_ (blue). For group *d* we assume that the logarithmic expression levels *x*_1_, *x*_3_, *x*_4_, *x*_5_, and *x*_6_ stem from the normal distribution with mean *μ*_0_ (red) and *x*_2_ stem from the normal distribution with mean *μ*_1_ (blue)

To illustrate this approach, we show the six measured logarithmic expression levels together with the univariate normal probability density estimated for group *a* and the three pairs of univariate normal probability densities estimated for each of the three groups $\tilde z \in \{b,c,d\}$ for gene *TPR* in Fig. 7. Visually, it is easy to see that the model of group *c* fits best the expression profile of this gene, as it yields the best separation between the two estimated means and the smallest estimated pooled variance. Consistent with this visual observation, the four corresponding likelihoods of the six measured logarithmic expression levels are *p*(*x*|*a*,*θ*_*a*_) =0.004, *p*(*x*|*b*,*θ*_*b*_)=0.035, *p*(*x*|*c*,*θ*_*c*_)=4.22, and *p*(*x*|*d*,*θ*_*d*_)=0.012, i.e., the likelihood of the six measured logarithmic expression levels of gene *TPR* is highest for group *c*.

However, performing classification through model selection based on maximizing the likelihood is problematic when the number of free model parameters is not identical among all models under comparison. In the BGSC approach, model *a* has two free model parameters, while models *b*, *c*, and *d* have three free model parameters. Hence, a simple classification based on maximizing the likelihood would give a spurious advantage to models *b*, *c*, and *d* with three free model parameters over model *a* with only two free model parameters. To eliminate that spurious advantage, we compute marginal likelihoods *p*(*x*|*z*) using the approximation of Schwarz et al. [14] commonly referred to as Bayesian Information Criterion (section “Probabilistic modeling of gene expression”). Applying this approximation to gene *TPR* we obtain the four marginal likelihoods of the six measured logarithmic expression levels *p*(*x*|*a*) = 0.001, *p*(*x*|*b*)=0.002, *p*(*x*|*c*)=0.287, and *p*(*x*|*d*)=0.001. We find that the marginal likelihood for group *c* is highest, which is consistent with the visual observation of Fig. 7.

To obtain the approximate posterior probability *p*(*z*|*x*), we now simply use Bayes’ formula *p*(*z*|*x*)=(*p*(*x*|*z*)*p*(*z*))/*p*(*x*) for group *z*∈{*a, b*,*c, d*}, where *p*(*z*) is the prior probability of group *z*, and the denominator *p*(*x*) is the sum of the four numerators *p*(*x*|*z*)*p*(*z*) for *z*∈{*a, b*,*c, d*}. We assume that 70% of all genes are not regulated by EGF, so we define the prior probability for group *a* by *p*(*a*)=0.70, and we further assume that the remaining 30% of the genes fall equally in groups with EGF-regulation, so we define the prior probabilities for groups *b*, *c*, and *d* by *p*(*b*)=*p*(*c*)=*p*(*d*)=0.1. Using these prior probabilities, we obtain for gene *TPR* the four approximate posterior probabilities *p*(*a*|*x*)=0.016, *p*(*b*|*x*)=0.008, *p*(*c*|*x*)=0.973, and *p*(*d*|*x*)=0.003. We find that the approximate posterior probability for group *c* is highest, so we finally assign gene *TPR* to group *c*.

By applying this approach of computing the four approximate posterior probabilities for each gene and assigning each gene to that group *z* with the highest approximate posterior probability, we classify 8449 genes to group *a*, 3822 genes to group *b*, 3143 genes to group *c*, and 1328 genes to group *d*.

### Prediction of genes belonging to simplified gene group *c*

For simplified gene group *c*, we define the subset of the 1140 genes with an approximate posterior probability *p*(*c*|*x*) exceeding 0.75 as putative target genes regulated by EGFR isoforms II-IV and not by other receptors (Additional file 2: Table S.1), and we scrutinize six of these genes in the following section. Three of these genes (*CKAP2L*, *ROCK1*, and *TPR*) are up-regulated with a log2-fold change $\hat {\mu }_{c1} - \hat {\mu }_{c0} >0.5$ and three of these genes (*ALDH4A1*, *CLCA2*, and *GALNS*) are down-regulated with a log2-fold change $\hat {\mu }_{c1} - \hat {\mu }_{c0} < -0.5$.

**Fig. 8:**
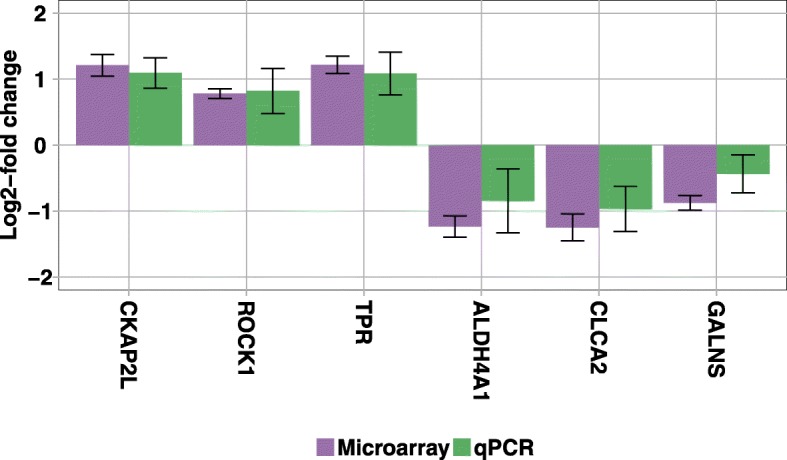
Comparison of microarray and qPCR log2-fold changes. Based on the microarray expression data described in Results, Discussion, Conclusions, and Methods we obtain an up-regulation for genes *CKAP2L*, *ROCK1*, and *TPR* and a down-regulation for genes *ALDH4A1*, *CLCA2*, and *GALNS*. The error bars are calculated using the Satterthwaite approximation. Based on the qPCR data, we obtain qualitatively and quantitatively similar results with overlapping error bars, yielding a Person correlation coefficient of the log2-fold changes of the microarray experiments and those of the qPCR experiments of 0.99

To validate the 36 logarithmic expression levels *x*_1_,…,*x*_6_ of the six genes *CKAP2L*, *ROCK1*, *TPR*, *ALDH4A1*, *CLCA2*, and *GALNS*, we perform 108 qPCR experiments comprising three biological replicates for each gene and each treatment. Figure 8 shows the 12 log2-fold changes $\hat {\mu }_{c1} - \hat {\mu }_{c0}$ of the microarray experiments and of the qPCR experiments. We find that the six log2-fold changes of the microarray experiments and those of the qPCR experiments are not identical, but in good agreement, yielding a Pearson correlation coefficient of 0.99. Moreover, the error bars, computed by using the Satterthwaite approximation, of all six genes overlap between microarray experiments and qPCR experiments.

To investigate the degree to which the expression levels of these genes respond to EGF in another glioblastoma cell line, we perform triplicated qPCR experiments in the glioblastoma cell line LNZ308 with and without EGF treatment. As *CLCA2* is not sufficiently expressed in cell line LNZ308 with a log-expression of −5.8 in the Cancer Cell Line Encyclopedia data [10], we stimulate cell lines SF767 and LNZ308 with EGF (50 ng/ml for 24 hours) and measure the expression of the five remaining genes by qPCR experiments. We find that the log2-fold changes are not identical, but in good agreement, between the two cell lines for the four genes *CKAP2L*, *ROCK1*, *TPR*, and *GALNS*, whereas they are different between the two cell lines for gene *ALDH4A1* (Additional file 1: Figure S.2).

## Discussion

### Adjustability of the EGFR-signaling pathway in cell line SF767

To analyze the function of the soluble EGFR (sEGFR) isoforms II-IV it is essential to use a cell line with an adjustable EGFR-signaling pathway. As shown in Fig. 1, the EGFR-signaling pathway is adjustable in cell line SF767 with respect to recombinant EGF stimulation, even though cell line SF767 has a *PIK3CA* (E545K) mutation resulting in a baseline level of AKT activation [15]. This mutation occurs in about 30% of human breast cancers, where it leads to gain-of-function mutations in gene *PIK3CA* that activate the PI3K-AKT-signaling pathway constantly, thereby uncoupling the EGFR response from AKT signaling [16]. However, in cell line SF767 the level of pAKT can be increased nearly three-fold in an EGF-dependent manner (Fig. 1) consistent with the observation of Sun et al. [17].

It has been suggested that glioblastoma cell lines with helical domain mutations are still sensitive to dual PI3Ki/MEKi treatment [9], which is consistent with our observation that the EGFR-signaling pathway is adjustable in cell line SF767. Also, it has been found that Gefitinib inhibited EGFR phosphorylation in U251MG and SF767 cells, whereas Gefitinib inhibited AKT phosphorylation only in SF767 cells but not in U251MG cells [18], consistent to Fig. 1. Other EGF-induced signaling pathways such as the PLC *γ*-signaling pathway appear to be intact in cell line SF767 too [19].

Next, we perform western blot experiments and find that both siRNAs reduce the levels of the full-length EGFR proteins (Fig. 2). By qPCR experiments we find that siRNA_*ALL*_ is capable of knocking down all *EGFR* splice variants and that siRNA_*I*_ is capable of selectively knocking down *EGFR* splice variant I (Additional file 1: Figure S.1). More precisely we detect a reduction by 70.9*%* on average for all *EGFR* splice variants and a reduction by 78.1*%* on average for *EGFR* splice variant I for siRNA_*ALL*_ as well as for siRNA_*I*_ (Additional file 1: Figure S.1). Based on similar reductions, it appears that *EGFR* splice variant I is the dominant splice variant. As expected, the level of *EGFR* splice variant IV was reduced only by siRNA_*ALL*_.

### Biological context of genes predicted to belong to simplified gene group *c*

Next, we investigate the biological context of the six genes predicted to belong to simplified gene group *c* by applying the BGSC approach under the simplifying assumption of neglecting the different binding affinities of the EGFR isoforms to EGF.

The ’Cytoskeleton Associated Protein 2 Like’ *(CKAP2L)* protein is localized on microtubules of the spindle pole throughout metaphase to telophase in wild-type cells [20], and a knock-down of *CKAP2L* has been found to suppresses migration, invasion, and proliferation in lung adenocarcinoma [21].

The ’Rho-Associated Protein Kinase 1’ *(ROCK1)* is known to play an important role in the EGF-induced formation of stress fibers in keratinocyte [22] and to be involved in the cofilin pathway in breast cancer [23]. Besides, ROCK1 has been found to promote migration, metastasis, and invasion of tumor cells and also to facilitate morphological cell shape transformations through modifications of the actinomyosin cytoskeleton [24].

Depletion of the mRNA of the ’Tumor Potentiating Region’ (*TPR*) gene by RNAi triggers G0-G1 arrest, and TPR depletion plays a role in controlling cellular senescence [25]. Also, TPR regulates the nuclear export of unspliced RNA and participates in processing and degradation of aberrant mRNAs [26], a mechanism considered important for the regulation of genes and their deregulation in cancer cells.

The ’Aldehyde Dehydrogenase 4 Family Member A1’ (*ALDH4A1*) gene contains a potential p53 binding sequence in intron 1, and *p53* is often mutated in tumor cells [27]. Moreover, *ALDH4A1* was induced in a tumor cell line in response to DNA damage in a p53-dependent manner [27], and depletion of the mRNA of *ALDH4A1* by siRNA results in severe inhibition of cell growth in HepG2 cells [28].

A second gene that is transcriptionally regulated by DNA damage in a p53-dependent manner is the ’Chloride Channel Accessory 2’ (*CLCA2*) gene. Inhibition of *CLCA2* stimulates cancer cell migration and invasion [29]. Furthermore, *CLCA2* could be a marker of epithelial differentiation, and knock-down of *CLCA2* causes cell overgrowth as well as enhanced migration and invasion. These changes are accompanied by down-regulation of E-cadherin and up-regulation of vimentin, and loss of *CLCA2* may promote metastasis [29]. Also, loss of breast epithelial marker *CLCA2* has been reported to promote an epithelial-to-mesenchymal transition and to indicate a higher risk of metastasis [30].

For the ’Galactosamine (N-Acetyl)-6-Sulfatase’ (*GALNS*) gene an effect of 17 *β*-estradiol on the expression of *GALNS* could be detected by qPCR experiments in a breast cancer cell line, which is a hint to a tumor association of *GALNS* [31].

Up-regulation of *ROCK1* and *TPR* and down-regulation of *ALDH4A1* and *CLCA2* (Fig. 8) are positively associated with the processes of migration, metastasis, and invasion of tumor cells and negatively associated with proliferation. The up-regulation of *CKAP2L* [32] by EGFR II-IV isoforms indicates a potential link to processes of cell-cycle progression of stem cells or progenitor cells. Overall, our interpretation of the impact of EGFR isoforms II-IV on four of six validated gene transcripts is that it seems likely that these isoforms are involved in processes of migration and metastasis of clonogenic (stem) cells, which is strongly associated with a more aggressive tumor and a worse prognosis of tumor disease.

We found that the BGSC approach was capable of detecting genes putatively regulated by EGFR isoforms II-IV and not by other receptors such as HER2, HER3, or HER4 [33], so we find it tempting to conjecture that the BGSC approach could be useful for the analysis of similarly-structured data of other nested experimental designs.

## Conclusions

We have performed RNAi experiments to analyze the expression of three poorly investigated isoforms II-IV of the epidermal growth factor receptor in glioblastoma cell line SF767 with an adjustable EGFR-signaling pathway, and we have developed the Bayesian Gene Selection Criterion (BGSC) approach for the prediction of putative target genes of these EGFR isoforms under the simplifying assumption of neglecting the different binding affinities of the EGFR isoforms to EGF. We have predicted 3143 putative target genes, out of which 1140 genes have an approximate posterior probability greater than 0.75, and we have tested six of these genes by triplicated qPCR experiments. These six genes include *ROCK1*, which is known to be associated with EGFR regulation, as well as *CKAP2L*, *TPR*, *ALDH4A1*, *CLCA2*, and *GALNS*. We have found that the six log2-fold changes of the microarray expression levels and those of the qPCR expression levels are highly correlated with a Pearson correlation coefficient of 0.99 (*p*-value = 0.00002), suggesting that the set of 1140 genes might contain some further putative target genes of EGFR isoforms II-IV in tumor cells. As suggested by our anonymous reviewers we like to point out that, in addition to RNAi, CRISPR/Cas knockout [34] and replacement with each isoform would be a promising strategy to discover additional functions of the soluble EGFR isoforms besides the ones described by Maramotti et al. [6]. The analysis of isoform-specific effects in combination with RNAi treatments are an elegant way to directly down-regulate specific mRNA splice variants, but that often leads to a nested experimental design for which generally no standard procedure exists. The two-step BGSC procedure of first defining easily interpretable conceptual groups of genes associated with different EGFR isoforms and subsequently classifying genes based on the approximated posterior probability to these groups seems to be a promising approach in such a situation, and this approach is readily adaptable to other and more complex experimental designs. The datasets analyzed during the current study and the R-scripts for reproducing the results and plots of this work are available in the BGSC repository, https://github.com/GrosseLab/BGSC.

## Methods

### Glioblastoma cell line SF767

We obtained glioblastoma cell line SF767 from Cynthia Cowdrey (Neurosurgery Tissue Bank, University of California, San Francisco, USA). We cultured cell line SF767 in RPMI1640 medium (Lonza, Walkersville, USA) containing 10% (Vol/Vol) fetal bovine serum, 1% (Vol/Vol) sodium pyruvate, 185 U/ml penicillin, and 185 *μ*g/ml ampicillin and maintain it at 37^∘^C in a humidified atmosphere containing 3% (Vol/Vol) CO_2_.

### Western blot and qPCR analyses

Cells were treated in lysis buffer, the protein concentration was determined using the Bradford method, and western blot analysis was performed as described in [35]. Antibodies directed against EGFR (Clone D38B1), HER2/ErbB2 (29D8), and phosphoserine 473 AKT (clone D9E) were obtained from Cell Signaling Technology Inc. (Signaling, Danvers, MA, USA), antibodies directed against *β*-actin were obtained from Sigma (Steinheim, Germany), and BIRC5 (Survivin) antibodies (clone AF886) were obtained from R&D systems (Richmond, CA, USA). qPCR experiments were performed as described in [35]. The primer sequences are listed in Table 3.

**Table 3:** Primer sequences for qPCR

Traget mRNA	Label	Sequence 5^′^→3^′^	Localization	Corresponding mRNA
ALDH4A1	Sense	AGTGGGACTTTGGCTGATCC	128-147	NM_170726.2
	Antisense	GTGAAGGCTAAGACGGGCTC	398-379	
CKAP2L	Dense	ACATCAGTGGAAGAGCTGGC	1940-1959	NM_152515.4
	Antisense	TTCTGCCTTGGCTATTCGGG	2044-2025	
CLCA2	Sense	CCATTGCCCTGGGTTCATCT	1690-1709	NM_006536.6
	Antisense	GGCCTGCCACGTAACTAGAA	1961-1942	
EGFR all	Sense	TCAGCCTCCAGAGGATGTTC	392-411	NM_005228.3
	Antisense	GTGTTGAGGGCAATGAGGAC	511-530	
EGFR v1	Sense	CCCAGTACCTGCTCAACTGG	2689-2709	NM_005228.4
	Antisense	TAGGCACTTTGCCTCCTTCTG	2889-2869	
EGFR v4	Sense	GCCATCCAAACTGCACCTAC	2105-2126	NM_201284.1
	Antisense	GGACACGCTGCCATCATTAC	2211-2192	
GALNS	Sense	CAGCTGTTGCTGGTGCTCAG	123-142	NM_000512.4
	Antisense	AGTTTGGGAAAAGCAGCCCT	303-284	
GAPDH	Sense	CACCCACTCCTCCACCTTTG	943-962	NM_002046.7
	Antisense	CCACCACCCTGTTGCTGTAG	1052-1033	
HPRT	Sense	TTGCTGACCTGCTGGATTAC	391-410	NM_000194.2
	Antisense	CTTGCGACCTTGACCATCTT	652-633	
MMP2	Sense	CCCTCGCAAGCCCAAGTGGG	650-669	NM_004530.5
	Antisense	CCATGCTCCCAGCGGCCAAA	848-828	
ROCK1	Sense	GGTGCTGGTAAGAGGGCATT	905-924	NM_005406.2
	Antisense	CGCAGCAGGTTGTCCATTTT	997-978	
TPR	Sense	GCTGAGGGTGGACTCGATTT	115-134	NM_003292.2
	Antisense	AGACTTGGGCAGCTTGTTCA	357-338	

### RNAi

The design and application of siRNA specific for EGFR mRNA and a nonsense siRNA were performed by a program provided by MWG (Eurofins Genomics, Ebersberg, Germany). The sequences of the double-stranded EGFR-specific siRNAs correspond to 21-bp sequences of the EGFR-cDNA (NCBI-ref NM _005228.3) for siRNA_*I*_ at positions 4094–4116 and for siRNA_*ALL*_ at positions 1258–1278 (Table 2). To ensure that the EGFR-specific siRNAs and the nonsense siRNA do not interact with other transcripts, we used the sequences of siRNA_*I*_, siRNA_*ALL*_, and nonsense siRNA to perform a BLAST search with Nucleotide BLAST against the human-genome database (http://www.ncbi.nlm.nih.gov/) and the siRNA-Check of SpliceCenter suite [36]. To prevent off-target effects of siRNA-treatment, we transfected cells with 50 *nM* targeting siRNA (siRNA_*I*_ and siRNA_*ALL*_) in RPMI complete medium. For transfecting we use the reagent INTERFERin™ according to the manufacturer’s instructions (Polyplus Transfection, Illkirch, France).

### Illumina BeadChip Microarray

RNA integrity and concentration were examined on an Agilent 2100 Bioanalyzer (Agilent Technologies, Palo Alto, CA, USA) using the RNA 6.000 LabChip Kit (Agilent Technologies) according to the manufacturer’s instructions. Illumina BeadChip analysis was conducted at the microarray core facility of the Interdisciplinary Center for Clinical Research (IZKF) Leipzig (Faculty of Medicine, University of Leipzig). 250 *ng* RNA per sample were ethanol precipitated with GlycoBlue (Invitrogen) as a carrier and dissolved at a concentration of 100–150 ng/ *μ*l before probe synthesis using the TargetAmp™- Nano Labeling Kit for Illumina Expression BeadChip (Epicentre Biotechnologies, Madison, WI, USA). 750 *ng* of cRNA were hybridized to Illumina HT-12 v4 Expression BeadChips (Illumina, San Diego, CA, USA) and scanned on the Illumina HiScan instrument according to the manufacturer’s specifications. The *read.ilmn* function of the *limma* package [37] was used to read the 47317 microarray probes into *R*. The *neqc* function of *limma* was used to perform a background correction followed by quantile normalization, using negative control probes for background correction and both negative and positive controls for normalization. The 16,742 array probes corresponding to 14,389 genes, which displayed a significant hybridization signal (Illumina signal detection statistic at *P*<0.05) in all probes were used for further analysis.

### Experimental design

For investigating which genes are activated by the four EGFR isoforms I - IV in glioblastoma cell line SF767 we use RNAi, as described in section “RNAi”, for a selective down-regulation of *EGFR* splice variants (Table 1 rows) with and without EGF treatment (Table 1 columns). Specifically, we applied the three different RNAi treatments – (i) control without RNAi, (ii) RNAi with siRNA_*I*_, and (iii) RNAi with siRNA_*ALL*_ – to glioblastoma cell line SF767.

In case (i) we performed a control experiment without RNAi treatment (Table 1, first row). Here, EGFR is not down-regulated by an siRNA, so target genes of all *EGFR* splice variants and other EGF receptors should be differentially expressed in columns 1 and 2, i.e., they should have different logarithmic expression levels *x*_1_ and *x*_2_.

In case (ii) we performed an RNAi with siRNA_*I*_, which can bind only to the full-length *EGFR* splice variant I (Table 1, second row). Hence, siRNA_*I*_ down-regulates splice variant I, but not the other splice variants II-IV, and in this case target genes of EGFR isoforms II-IV and of other EGF receptors should be differentially expressed in columns 1 and 2, i.e., they should have different logarithmic expression levels *x*_3_ and *x*_4_.

In case (iii) we performed an RNAi with siRNA_*ALL*_, which can bind to all four *EGFR* splice variants, and subsequently down-regulates all four splice variants (Table 1, third row). Here, only target genes of other EGF receptors should be differentially expressed in columns 1 and 2, i.e., they should have different logarithmic expression levels *x*_5_ and *x*_6_.

### Probabilistic modeling of gene expression

We propose a probabilistic model for the logarithmic expression pattern *x*=(*x*_1_,…,*x*_6_) for each of the four groups *z*∈{*a, b*,*c, d*} defined in section “First step of the BGSC approach - grouping of genes”.

First, we assume that the three logarithmic expression levels *x*_1_, *x*_3_, and *x*_5_ corresponding to no EGF treatment are similar to each other, which corresponds to the assumption that the RNAi treatment should have no effect in case of no EGF treatment. Second, we assume that the three logarithmic expression levels *x*_2_, *x*_4_, and *x*_6_ follow the expression patterns described in section “First step of the BGSC approach - grouping of genes” and summarized in Fig. 5.

In order to mathematically formulate the model assumptions, we introduce six indicator variables *g*_1_,…,*g*_6_ for the groups $\tilde z \in \{b, c, d\}$ that indicate if the six logarithmic expression levels *x*_1_,…,*x*_6_ are expected to be different from *x*_1_. Specifically, we define *g*_*n*_=1 if *x*_*n*_ is expected to be different from *x*_1_ for *n*=1,…,6 and *g*_*n*_=0 otherwise. Genes of group *a* are defined as showing no effect on the EGF treatment and therefore *g*_*n*_ equals 0 by definition.

By definition, we obtain that *g*_1_=0 for each of the three groups $\tilde z$. From the first model assumption we obtain that *g*_1_, *g*_3_, and *g*_5_ are equal to 0 for each of the three groups $\tilde z$. From the second model assumption we obtain that (*g*_2_,*g*_4_,*g*_6_) is equal to the corresponding column of Fig. 5 for each of the three groups $\tilde z$. Figure 6 summarizes the values of the indicator variables *g*_1_,…,*g*_6_ for each of the three groups *b*−*d*.

Third, we assume that the logarithmic expression levels *x*_1_,…,*x*_6_ are statistically independent and normally distributed. By combining all three model assumptions, we obtained the likelihood

1 $$\begin{array}{*{20}l}  p(x | a, \theta_{a}) &= \prod_{n=1}^{6} \mathcal{N} (x_{n} | \mu_{a}, \sigma_{a}) \end{array} $$

2 $$\begin{array}{*{20}l}  p(x | \tilde z, \theta_{\tilde z}) &= \prod_{n=1}^{6} \mathcal{N} (x_{n} | \mu_{\tilde z g_{n}}, \sigma_{\tilde z}) \end{array} $$

for each of the four gene groups *z*∈{*a, b*,*c, d*}, where

3 $$\begin{array}{*{20}l} \mathcal{N} (x_{n} | \mu_{a}, \sigma_{a}) &= \frac{1}{\sqrt{2\pi}\sigma_{a}} ~ \times ~ e^{- \frac{ (x_{n}- \mu_{a})^{2}}{2\sigma_{a}^{2}}} \end{array} $$

denotes the density of the normal distribution, *θ*_*a*_=(*μ*_*a*_,*σ*_*a*_) denotes the parameter of model *a*, and

4 $$\begin{array}{*{20}l} \mathcal{N} (x_{n} | \mu_{\tilde z g_{n}}, \sigma_{\tilde z}) &= \frac{1}{\sqrt{2\pi}\sigma_{\tilde z}} ~ \times~ e^{- \frac{ (x_{n}- \mu_{\tilde z g_{n}})^{2}}{2\sigma_{\tilde z}^{2}}} \end{array} $$

denotes the density of the normal distribution, $\theta _{\tilde z} = (\mu _{\tilde {z}0}, \mu _{\tilde {z}1}, \sigma _{\tilde z})$ denotes the parameter of model $\tilde z$, and *g*_*n*_ are the indicator variables from Fig. 6.

### Posterior approximation by the Bayesian Information Criterion

Next, we seek the approximate posterior

5 $$\begin{array}{*{20}l}  p(z|x) &= \frac{ p(x|z) p(z)} {p(x)} \end{array} $$

for each *z*∈{*a, b*,*c, d*} and each gene, where *p*(*z*) is the prior probability of group *z*.

For the four models of section “Probabilistic modeling of gene expression” the approximations of the marginal likelihoods based on the Bayesian Information Criterion are

6 $$\begin{array}{*{20}l}  p(x|z) &\propto \frac{p(x| z, \hat \theta_{z})}{ \sqrt{6}^{|\theta_{z} |} }, \end{array} $$

where 6 is the number of data points and |*θ*_*z*_| is the number of free parameters of model *z*, which is 2 for group *a* and 3 for groups *b*−*d*, and where the maximum-likelihood estimators $\hat \theta _{z}$ are

8a $$\begin{array}{*{20}l}  {}\hat{\mu}_{a} &= \frac{1}{6} \sum_{n=1}^{6} x_{n}  \end{array} $$

8b $$\begin{array}{*{20}l}  {}\hat{\sigma}_{a}^{2} &= \frac{1}{5} \sum_{n=1}^{6} (x_{n}- \hat{\mu}_{a})^{2}  \end{array} $$

8c $$\begin{array}{*{20}l}  {}\hat{\mu}_{\tilde z0} &= \frac{ \sum\limits_{n=1}^{6} x_{n} (1-g_{\tilde zn}) }{\sum\limits_{n=1}^{6} (1-g_{\tilde zn})} \end{array} $$

8d $$\begin{array}{*{20}l}  {}\hat{\mu}_{\tilde z1} &= \frac{ \sum\limits_{n=1}^{6} x_{n} g_{\tilde zn} }{\sum\limits_{n=1}^{6} g_{\tilde zn}}  \end{array} $$

8e $$\begin{array}{*{20}l}  {}\hat{\sigma}_{\tilde z}^{2} &= \frac{ \displaystyle \sum_{n=1}^{6} (x_n- \hat{\mu}_{\tilde z0})^{2} (1-g_{\tilde zn}) + \sum_{n=1}^{6} (x_n- \hat{\mu}_{\tilde z1})^{2} g_{\tilde zn} }{4}  \end{array} $$

for $\tilde z \in \{b, c, d\}$, and where $g_{\tilde zn}$ denotes the indicator variable *g*_*n*_ of group $\tilde z$. Based on these approximations, we compute *p*(*z*|*x*) and then perform Bayesian model selection by assigning each gene to that group *z* with the maximum approximate posterior *p*(*z*|*x*).

## Additional files

Additional file 1 Figure S.1 Expression of EGFR splice variants, GAPDH, and MMP2
Figure S.2 log2-fold changes of the qPCR expression levels for cell lines SF767 and LNZ308. (PDF 96 kb)

Additional file 2 Table S.1. Predicted genes belonging to simplified gene group *c*. (XLSX 411 kb)

## Data Availability

The datasets analyzed during the current study are available in the BGSC repository, https://github.com/GrosseLab/BGSC/.

## Abbreviations

AKT: Serine-threonine protein kinase
ALDH4A1: Aldehyde Dehydrogenase 4 family member A1
AREG: Amphiregulin
BGSC: Bayesian Gene Selection Criterion
BIRC5: Baculoviral IAP repeat containing 5
CKAP2L: Cytoskeleton associated protein 2 like
CLCA2: Chloride channel accessory 2
CPCC: CKAP2-positive cell count
EGF: Epidermal growth factor
EGFR: Epidermal growth factor receptor
GALNS: Galactosamine (N-Acetyl)-6-Sulfatase
GAPDH: Glyceraldehyde-3-Phosphate Dehydrogenase
HER2: Human epidermal growth factor receptor 2
HPRT: Hypoxanthine Phosphoribosyltransferase 1
MMP2: Matrix Metallopeptidase 2
PI3K: Phosphatidylinositol 3-kinase
PIK3CA: Phosphatidylinositol 3-Kinase catalytic subunit alpha
PLC *γ*: Phospholipase C gamma
PTEN: Phosphatase and tensin homolog
qPCR: Quantitative real-time polymerase chain reaction
RNAi: RNA interference
ROCK1: Rho-associated protein kinase 1
sEGFR: Soluble EGFR
siRNA: Small interfering RNA
TGF *α*: Transforming growth factor alpha
TKI: Tyrosine kinase inhibitors
TPR: Tumor potentiating region
