# Supplementary material for: Prediction of regulatory targets of alternative isoforms of the epidermal growth factor receptor in a glioblastoma cell line
Source: BMC Bioinformatics. 2019 Aug 22;20:434. doi: 10.1186/s12859-019-2944-9 (PMC6704634; doi:10.1186/s12859-019-2944-9)
Supplement: Supplementary file 1 — Figure S.1 Expression of EGFR splice variants, GAPDH, and MMP2 Figure S.2 log2-fold changes of the qPCR expression levels for cell lines SF767 and LNZ308. (PDF 96 kb) [file 12859_2019_2944_MOESM1_ESM.pdf]

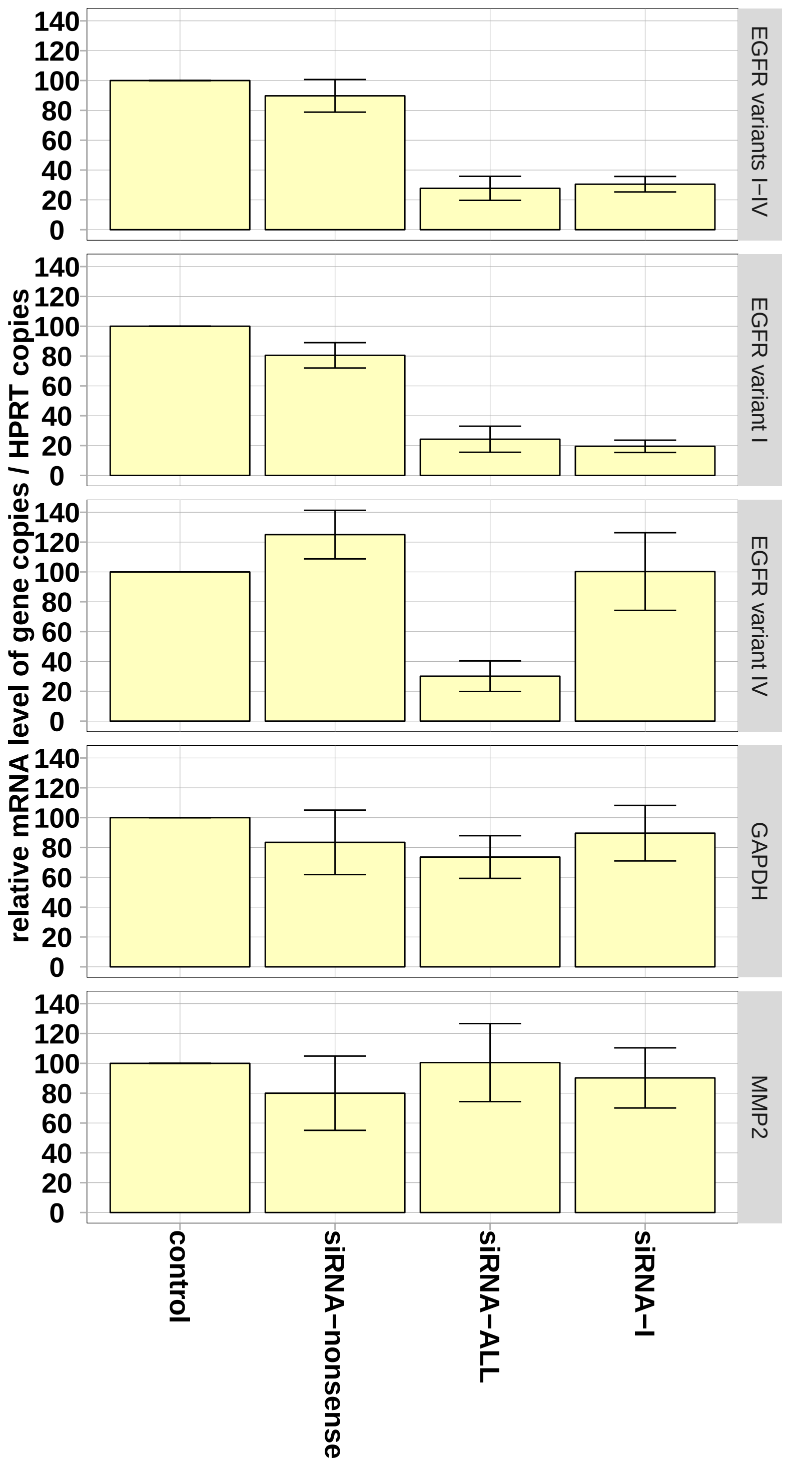

Figure S.1: **Expression of *EGFR* splice variants, *GAPDH* and *MMP2*** 48 hours after the application of three siRNA constructs ($siRNA_{nonsense}$ as control, $siRNA_{ALL}$, $siRNA_{I}$) the mRNA levels of all *EGFR* splice variants, full-length EGFR (*EGFR* splice variant I), *EGFR* splice variant IV, *GAPDH*, and *MMP2* were measured and normalized to *HPRT*. The analysis was performed with at least three independent experiments in the cell line SF767. Compared to the control experiments the $siRNA_{ALL}$ and the $siRNA_{I}$ reduced the levels of mRNA of all *EGFR* splice variants by about 70% and of *EGFR* splice variant I by about 75%. The *EGFR* splice variant IV was reduced only by the siRNA $siRNA_{ALL}$ and $siRNA_{I}$ did not affect the mRNA level. As expected, compared to the control experiment the mRNA levels of *GAPDH* and *MMP2* are not reduced.

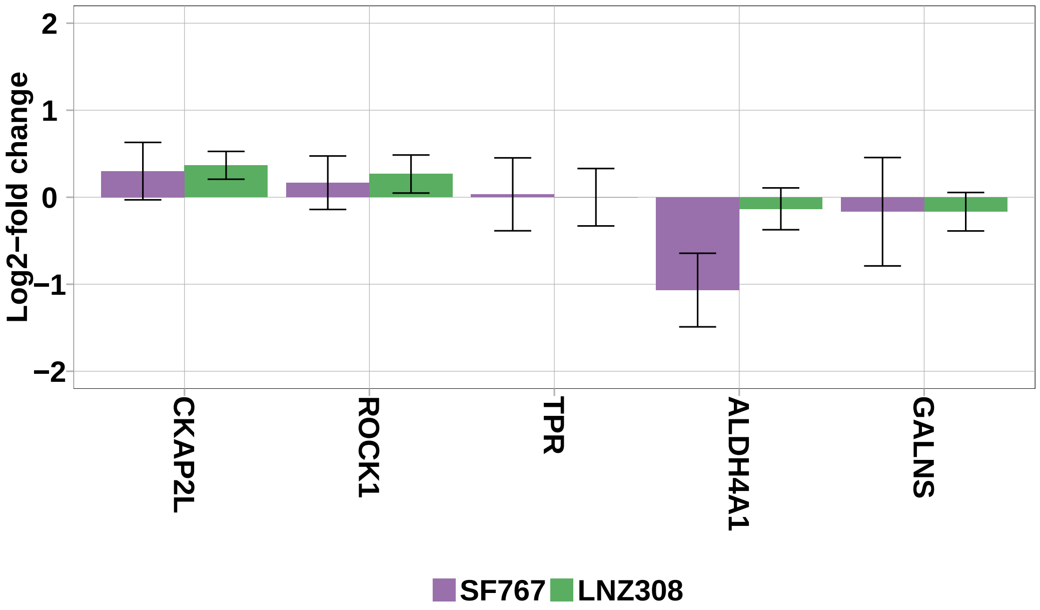

Figure S.2: $\log_2$**-fold changes of the qPCR expression levels for cell lines SF767 and LNZ308** The cell lines (SF767 and LNZ308) were stimulated with the ligand EGF (50ng/ml for 24 hours) and the gene expressions of *CKAP2L*, *ROCK1*, *TPR*, *ALDH4A1* and *GALNS* were measured by qPCR experiments. The analysis were performed with at least three independent experiments for each cell lines. The $\log_2$-fold changes of the qPCR experiments were calculated for control without EGF vs control with EGF stimulation based on the $\Delta\Delta CT$-values compared to GAPDH. By the comparison of $\log_2$-fold changes of the cell lines SF767 and LNZ308, we could validate four genes (*CKAP2L*, *ROCK1*, *TPR*, and *GALNS*) by having overlapping error bars and detect that ALDH4A1 was differently down-regulated in both cell lines.
